# Feeding Experiences Among Caregivers of Children With Down Syndrome in Rwanda: A Qualitative Exploratory Study

**DOI:** 10.1002/puh2.70230

**Published:** 2026-04-10

**Authors:** Joselyne Rugema, Eric Matsiko, Vedaste Bagweneza, Donatilla Mukamana, Leon Mutesa

**Affiliations:** ^1^ College of Medicine and Health Sciences, School of Nursing and Midwifery University of Rwanda Kigali Rwanda; ^2^ College of Medicine and Health Sciences, School of Public Health University of Rwanda Kigali Rwanda; ^3^ College of Medicine and Health Sciences, School of Medicine and Pharmacy University of Rwanda Kigali Rwanda; ^4^ Rwanda Military Referral and Teaching Hospital Kigali Rwanda; ^5^ College of Medicine and Health Sciences, Center of Human Genetics University of Rwanda Kigali Rwanda

**Keywords:** caregivers, children, Down syndrome, feeding, nutrition, Rwanda

## Abstract

**Background and Aims:**

Down syndrome (DS) affects approximately 1 in 700 to 1000 live births globally. Children with DS often face feeding difficulties that arise from low muscle tone and oral‐motor dysfunction, making complementary feeding initiation particularly challenging. Despite the role of caregivers in addressing these challenges, research on their feeding patterns in low‐resource settings like Rwanda is scarce. Therefore, we aimed at exploring the feeding patterns of caregivers of children with DS in Rwanda.

**Methods:**

We employed a qualitative exploratory design involving 34 caregivers from four hospitals. Participants were recruited through convenience sampling. Twenty‐eight people participated in focus group discussions (FGDs), while six took part in in‐depth interviews (IDIs). The level of saturation determined the number of FGDs and IDIs. Informed consent was obtained before participation, and data collectors were granted permission to audio‐record the interviews. The collected data were subsequently transcribed and analyzed thematically.

**Results:**

Caregivers reported different developmental delays in children with DS, affecting timely initiation and progression of complementary feeding. Feeding challenges were more pronounced in children with DS compared to those without, with delays in food introduction and age‐related changes in food consistency. Financial constraints and anatomical anomalies further contributed to difficulties in adequately feeding children with DS. Participants reported receiving necessary support for caring for their children from local authorities, healthcare facilities, and family members. However, some participants reported that male partners did not provide adequate support in caring for their children, with some husbands even abandoning the household following the diagnosis of DS.

**Conclusion:**

Caregivers of children with DS indicated that these children have developmental delays that negatively impact their feeding. They emphasized that financial issues and their anatomical anomalies contribute to feeding difficulties. Many participants also noted that insufficient support from male partners hinders their ability to provide proper nutrition.

## Graphical abstract

In Rwanda, Caregivers of children with Down Syndrome face multiple feeding challenges, like development delays and physiological changes of their children, financial limitations, and insufficient partner support, they adapt via increasing frequency meal, time intensive care which promote bonds through love and faith, Additionally They recommend Health Support Systems.

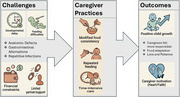

**Abbreviations**
DS: Down syndrome
FGD: focus group discussion
IDI: in‐depth interview

## 1 Introduction

Congenital disorders can cause long‐term disabilities, placing a significant burden on individuals, families, healthcare systems, and society. Among the most common and severe congenital conditions are heart defects, neural tube defects, and chromosomal abnormalities such as Down syndrome (DS) [1]. Chromosomal abnormalities are increasingly prevalent and represent the most common cause of congenital abnormalities in humans. Statistics indicate that the total estimated prevalence of chromosomal abnormalities ranges from 48 to 90 per 10,000 births, depending on factors such as race, observation period, and regional differences [2]. DS is the most common chromosomal abnormality, and it typically arises when an individual has an extra copy of chromosome 21. This condition affects approximately 1 in every 700 to 1000 live births globally [3, 4, 5]. There are substantial variations in the burden and trends of DS across different regions and countries, influenced by their unique sociodemographic characteristics. However, in regions with a low sociodemographic index, the number of prevalent DS cases has been rising, which raises serious concerns [4].

Children with DS exhibit distinct physical characteristics, experience delays in psychomotor development, and have a higher likelihood of congenital malformations [6]. However, there is significant individual variability; some children experience mild symptoms and complications, whereas others are more profoundly affected. This variability also extends to their risk of developing nutrition‐related health issues, as their other health challenges can impact nutritional aspects [3, 7]. Gastrointestinal (GI) alterations, which are common in children with DS, primarily contribute to these nutrition‐related challenges [8].

Previous studies have shown that children with DS are susceptible to a wide range of GI alterations, which can significantly impact their feeding, nutritional status, and overall growth. These alterations may present as symptoms such as vomiting, diarrhea, abdominal pain, and constipation [9, 10]. In addition, children with DS may experience developmental delays that contribute to feeding difficulties, as well as congenital structural anomalies, including duodenal atresia, imperforate anus, and esophageal atresia [9, 11].

Therefore, careful attention to nutritional intake is crucial, as various characteristics and comorbidities associated with DS can have significant nutritional implications and consequences [12].

Children with DS often face greater difficulties with milk feeding and transitioning to solid foods compared to typically developing children. For example, a study conducted by Anil et al. in India highlighted difficulties across all three phases of swallowing, with the greatest challenges occurring when eating solids, followed by liquids [13]. The same study indicated that children with DS faced issues related to the physical, functional, and emotional aspects of feeding. Moreover, that study revealed that children with DS exhibited impaired oro‐sensorimotor abilities, which likely contributed to their feeding difficulties [13]. A scoping review of 18 studies revealed that children with DS tend to be introduced to complementary foods later than their typically developing peers and experience slower progression toward more complex food textures. Delays in both gross and fine motor skills, as well as sensory challenges, contribute to secondary feeding difficulties, including challenges with chewing, biting, and reduced awareness of food on their lips and tongue [11], making the feeding patterns of their caregivers a vital area of study, especially in the Rwandan context. In our study context, the term “caregivers” refers to parents, guardians, and other family members taking care of children with DS, as they play a critical role in managing different challenges children with DS may face in their daily living and ensuring the child's nutritional needs are met, particularly during the initiation of complementary feeding and beyond.

Parents and caregivers face challenges in feeding children with DS because of the health issues that are typically unique for these children. Parents of children with DS tend to be more cautious and adopt more controlling feeding practices compared to those with typically developing children. They also express greater concern about their child's weight [11]. Caregivers express heightened stress related to feeding their child with DS. The primary sources of this stress include worries about whether their child is eating enough, and the challenges associated with feeding difficulties. Caregivers also express greater stress when their child is learning a new feeding skill or transitioning to a different feeding phase. To cope with these challenges, they rely on professional support, personal networks, and a combination of problem‐solving and emotion‐focused strategies [14].

There is a scarcity of studies exploring feeding patterns among caregivers of children with DS, particularly in low‐resource settings like Rwanda, where this issue remains underexplored. This limited research may be attributed to the fact that genetic disorders are not widely recognized or understood in the community and the marginalization of individuals with DS, a trend commonly observed in lower income countries such as Rwanda [15, 16], despite a study conducted in Rwanda indicating that DS is the most common chromosomal abnormality [17]. As a result, this important aspect of care has not been fully investigated. Given the limited studies exploring feeding patterns for children with DS, understanding these patterns among caregivers is particularly essential.

In Rwanda, various factors, such as the sociocultural context and economic conditions, influence how caregivers manage the feeding needs of children [18]. This phenomenon is not unique to Rwanda; existing evidence from other contexts also indicates that caregivers’ socioeconomic status, childcare setting, educational background, cultural beliefs, and employment status can significantly affect food availability, parental feeding practices, and access to professional feeding support [11]. However, despite this well‐established evidence in the general population, there is a notable scarcity of studies exploring the experiences of caregivers of children with DS regarding how these factors influence their feeding practices for their children.

Therefore, this study aimed to address this gap by exploring the feeding experiences among caregivers of children with DS in Rwanda. The study sought to uncover the practices, challenges, and strategies employed by caregivers to cope with these challenges, providing insights that could inform future interventions and support services, ultimately improving the nutritional outcomes for children with DS in Rwanda.

## 2 Methods

### 2.1 Study Setting

The study was conducted at two university teaching hospitals in Kigali City, namely, Rwanda Military Referral and Teaching Hospital and the Centre Hospitalier Universitaire de Kigali, and two district hospitals in the Eastern Province, which are Kirehe and Rwinkwavu hospitals. The university teaching hospitals were chosen because they offer genetic services, have specialists in genetics, and possess appropriate means for accurately diagnosing DS. The two district hospitals were selected due to their partnership with a non‐governmental organization, Partners in Health (Inshuti Mu Buzima), which supports children with disabilities, including those with DS. Their selection ensured access to caregivers of children with a confirmed diagnosis of DS.

### 2.2 Study Design

In this study, we employed qualitative exploration design using in‐depth interviews (IDIs) and focus group discussions (FGDs).

### 2.3 Study Population, Sample Size, and Participants Recruitment

The study population comprised caregivers of children with DS. A total of 34 caregivers were recruited using a convenience sampling strategy. The convenience sampling was employed because the data collectors met participants at study settings when they have attended them for usual services. To be included in the study, participants had to have a child with DS and to voluntarily consent to participate. For the purposes of this study, a child was defined as an individual with DS under 18 years of age, in line with internationally accepted definitions of childhood [19]. Selection ensured representation from various study settings, including rural areas in the Eastern Province and urban areas in the City of Kigali. The number of FGDs and IDIs was determined based on the point of data saturation.

### 2.4 Data Collection Procedure and Tools

Two members of the research team were directly involved in data collection, along with two additional trained data collectors under the supervision of the principal investigator (PI). These trained data collectors were selected on the basis of their experience in qualitative data collection and their professional backgrounds as either nurses or midwives previously involved in similar fieldwork. This careful selection process helped ensure the reliability and credibility of the data collected. During data collection, each interview or FGD was conducted by two individuals: one serving as the primary interviewer and the other as the note‐taker. Among the four data collectors, two were male and two were female.

Data collectors first obtained participants’ consent to take part in the study by providing a detailed explanation of the study's purpose, potential benefits, and risks. As the study involved recording interviews, they then sought additional permission to record. A semi‐structured interview guide, consisting of 11 open‐ended questions, was used to gather information through FGDs and IDI. The development of the guide was informed by a review of the literature. Each FGD comprised 7, and a total of 4 FGDs were conducted. Three FGDs were made of female participants, whereas one FGD was made of male participants. Additionally, six IDIs were conducted. The participants in both FGDs and IDIs were the caregivers of children with DS.

Triangulation is defined as using multiple data sources, methods, or theoretical perspectives to study a single phenomenon, which subsequently increases the credibility and validity of research findings [20]. This qualitative study used method triangulation by combining FGDs and IDIs for data collection, as each method offered unique insights that could not be captured by using only one. IDIs were conducted first with participants at each study setting, followed by one FGD at the same site.

This approach allowed the study to capture individual experiences in depth through IDIs and then explore collective perspectives in FGDs, identifying areas of agreement, diversity, or differing opinions within participant groups. IDIs were especially valuable for understanding personal and sensitive experiences, such as those related to having a child with DS. FGDs, on the other hand, created a supportive environment where participants who were hesitant to speak individually could share their thoughts, and where they could feel reassured and encouraged by realizing they were not alone in facing challenges related to feeding and raising children with DS [20].

Data collection took place in March and April 2024. The average duration for each group discussion was 1 h, while the average duration for each IDI was 30 min. Data collection was conducted in Kinyarwanda, a local language that most participants could understand, and this allowed them to express their ideas freely and appropriately. All resulting transcripts were translated into English for analysis, report, and manuscript writing.

### 2.5 Ethical Considerations

The researcher adhered to ethical standards for studies involving human participants. Ethical clearance was obtained from the University of Rwanda's Institutional Review Board (Approval No. 482/CMHS IRB/2023), and official permission was secured from all study sites. Participants provided informed consent for participation and audio recording, with the assurance that they could withdraw at any time without any negative consequences. Given the sensitivity of topics related to DS, interviews were conducted in a safe and respectful environment, with psychological support available if needed. Confidentiality and anonymity were strictly maintained, as no identifying information was included in transcripts or reports. Data, including audio recordings and transcripts, were securely stored on the PI's password‐protected computer and used solely for academic purposes such as publication or stakeholder dissemination.

### 2.6 Data Analysis

A thematic analysis approach was employed to analyze the qualitative data using ATLAS.ti software.

A specialist transcriber transcribed the interviews verbatim in Kinyarwanda. The research team members examined the transcripts and field notes, establishing the codebook based on emerging ideas recorded during the analysis.

The data analysis began concurrently with data collection. There was a continuous iterative process between data collection and analysis, with the researchers immersing themselves in the data through repeated listening to audio recordings and reading collected materials to gain a deeper understanding. Crucial phrases and statements were extracted from the transcribed data, and a list of meanings was generated through repeated review and reflection on participants’ contributions.

The PI and some research team members independently coded the data. They then compared their coded data to identify any discrepancies or agreements and reached a consensus on the final coding. The double coding process ensured consistent data coding, enhancing the validity of the research findings. Findings were presented using themes and supporting quotes.

### 2.7 Techniques to Enhance Trustworthiness

Researchers implemented key processes to ensure the trustworthiness of the findings. These processes included conducting a peer review within the research team prior to paper submission, ensuring data saturation, assessing the strengths and limitations of the study, and providing detailed descriptions of the study's context, participants, and methodology. Given that this manuscript is part of the doctoral study of the PI, supervisors supported the maintenance of a reflective journal and encouraged engagement in discussions regarding the PI's and the data collectors’ positionalities. Their guidance was instrumental in helping the PI and data collectors critically evaluate how their personal experiences and social identities might influence interactions with participants and the interpretation of data. This reflective practice significantly contributed to enhancing the integrity and trustworthiness of the study findings.

In addition, the trustworthiness of the study was strengthened during the translation process. A native Kinyarwanda speaker who is also fluent in English translated the transcripts into English, and a second translator performed a back‐translation. Neither translator was involved in the data collection process. The back‐translation closely matched the initially translated version, ensuring the accuracy and consistency of the translation. Throughout the transcription and translation process, the research team meticulously double‐checked to verify the accuracy of both the transcription and translation.

## 3 Results

### 3.1 Sociodemographic Characteristics of Participants

Table 1 presents the sociodemographic characteristics of the participants. Out of 34 individuals who took part in the study, the majority were female (76%, *n* = 26), whereas only 8 (24%) were male. Regarding the participants’ age, the majority were between 41 and 50 years old (53%, *n* = 18), whereas a small proportion were aged 51–60 years (9%, *n* = 3). In terms of education level, most participants had completed primary education (47%, *n* = 16). The remaining 53% comprised those who had never attended school (35%, *n* = 12) or had attended secondary education (18%, *n* = 6).

**TABLE 1:** Sociodemographic characteristics of participants.

SD characteristics	*n*	Percentage
**sex of participants**		
Female	26	76
Male	8	24
**Age range of participants**		
20–30 years	4	12
31–40 years	9	26
41–50 years	18	53
51–60 years	3	9
**Level of education**		
No education	12	35
Primary education	16	47
Secondary education	6	18
**Marital status**		
Single	1	3
Married	13	38
Divorced	1	3
Separated	18	53
Widowed	1	3
**Occupation of participants**		
No occupation	5	15
Farmer	26	76
Private job or business	2	6
Public servant	1	3

Concerning marital status and occupation, most participants (53%, *n* = 18) were separated, and most of them were farmers (76%, *n* = 26), with only one participant working as a public servant.

### 3.2 Feeding Experiences Among Caregivers of Children With DS

Table 2 displays four main themes that emerged from the thematic analysis: (a) feeding experiences and practices for children with DS; (b) parental motivation for feeding children with DS; (c) support systems and resources for feeding children with DS; (d) challenges related to feeding children with DS.

**TABLE 2:** Themes and subthemes.

Themes	Subthemes
Feeding experiences and practices for children with Down syndrome	Delayed readiness of children for complementary feeding
	Developmental delays contributing to delays in food introduction Changes in food consistency based on child's development Feeding experiences of children with and without Down syndrome
Parental motivation for feeding children with Down syndrome	Love and respect for the child as a creation of God
	Witnessing the tangible growth of the child
Support systems and resources for feeding children with Down syndrome	Support from family, the community, government, and healthcare providers
	Family support in caregiving to children with Down syndrome
Challenges related to feeding children with Down syndrome	Financial influences on food choices
	Anatomical anomalies contribute to feeding difficulties Lack of support from male partners

#### 3.2.1 Theme 1: Feeding Experiences and Practices for Children With DS

Participants shared diverse experiences regarding the feeding of children with DS. They particularly emphasized the challenge of introducing complementary feeding, noting that children with DS often exhibit delays in readiness for such feeding. This delay is frequently attributed to different anomalies commonly observed in children with DS, such as oral motor difficulties that impede proper chewing and swallowing, which are typically expected around the 6‐month mark. Additionally, they highlighted the increased frequency of feeding required for these children compared to those without DS as a means to address and compensate for these feeding challenges.

Participants in both IDIs and FGDs also provided diverse perspectives regarding this theme, focusing on types of foods used, changes made as children grew, and feeding experiences of children with and without DS.

##### 3.2.1.1 Delayed Readiness of Children for Complementary Feeding

Participants who participated in the study shared their insights on the readiness of their children for complementary feeding at 6 months, the majority explaining that their children were not ready at that age, which often led to a delayed introduction of complementary feeding. Participants noted that they coped by gradually encouraging and sometimes gently forcing their children until they were able to swallow complementary food. Only a few participants indicated that their children were ready for complementary feeding at 6 months.

Starting from six months, I was giving him food and saw that he did not want it, and he did not know how to eat it. Even the porridge we had to pour it into his mouth; he did not know how to take it. I had to give him soft foods, but he was not eager to eat them. He did not have any courage to eat it. I kept on trying with the porridge, but he could not eat the food. So, I left it and was only giving him porridge, and when he would take two tablespoons of porridge, it was enough. I then stopped breastfeeding him. **(FGD, Female participant 5, Eastern Province)**

My child was not really ready for complementary feeding at six months, she had no courage to put in the mouth like other children. I introduced complementary feeding as drugs because she could not open her mouth, but I had to try and put it in her mouth to make her familiar. I decided to introduce it because I knew that a child starts taking complementary feeding at six months. **(FGD, Female Participant 4, Eastern Province)**.

At 6 months, my child was ready because I observed that breastmilk was no longer enough to satisfy him. Then, that is why I decided to initiate complementary feeding and keep breastfeeding him, it brought some improvements. **(FGD, Female Participant 6, Eastern Province)**.

##### 3.2.1.2 Developmental Delays Contributing to Delays in Food Introduction

Some participants expressed that those developmental delays in children with DS contributed to delays in the food introduction. As a result, they often had to attempt feeding their children multiple times a day to compensate for difficulties with food intake and the children's reluctance to eat.

I introduced this child to complementary feeding at 8 months. At 8 months they could even feed themselves, but for my child who has DS, it was challenging because the child got a year without being able to chew or swallow. Others at one year could feed themselves with visible appetite, but for mine, it was very challenging to eat solid foods. For this child I could even feed him more often compared to others because I wanted to compensate. I could feed this child 6 times a day while it was 3 times for the normal ones. **(IDI, Female participant, Eastern Province)**

A child with DS must be fed more times than another child without DS. He cannot tell you that he is hungry, you must observe him and know when to feed him. When he is often crying or behaves differently, it may be a sign that he is hungry, and you have to feed him. **(FGD, Female Participant 5, Eastern Province)**

##### 3.2.1.3 Changes in Food Consistency Based on Child's Development

Progressive changes in food consistency were noted, with children with DS first being introduced to semi‐solid foods and later transitioning to solid foods as they developed. However, the main issue reported was that many children delayed transitioning to age‐appropriate foods; some did not begin eating semisolid foods until the age of five, which is uncommon for children without DS. Participants mentioned that they considered the child's ability to eat different types of foods and made necessary adjustments.

As the child was growing, we introduced some semi‐solid and solid food to strengthen her jaws. In the beginning, she could not even eat a banana fruit without softening it, but now she can eat it, and I also feed her with different tubers. **(IDI, Female Participant, Kigali City)**

My child started eating at 9 months, we started with cow's milk, and we introduced soft fruits. Those are the foods he started eating, just soft foods. We did not introduce solid food immediately. Before 9 months, he did not even know about the food. He did not have appetite for it, he could even vomit it. **(FGD, Male Participant 6, Eastern Province)**.

I started feeding him with highly softened food for up to four years. From five years, I tried to feed him with semi‐solid foods, and I found it became more helpful, and even his stomach became ready to manage those foods. **(IDI, Female participant, Kigali City)**

I started giving food to my child when I weaned him. He only lived on breast milk, and it did not really help in his growth. He did not gain weight like other children; he did not have a strong body; he was really stunted because of feeding breastmilk alone. When I weaned him from breastmilk and started giving him real food and porridge, he started growing. **(FGD, Male participant 2, Eastern Province)**

##### 3.2.1.4 Feeding Experiences of Children With and Without DS

Participants shared different perspectives on the experiences of feeding children with DS compared to those without. They mentioned that, due to the difficulties these children face, careful attention is needed to ensure they can either feed themselves or still require assistance. It was also reported that children with DS often do not express hunger, making it the responsibility of caregivers to ensure they are fed regularly. Despite not showing signs of hunger, they still need to be fed consistently, as they often experience hunger more frequently than children without DS.

Children with DS and those without DS are different. A child with DS must be fed more times than another child without DS. **(FGD, Female participant 3, Eastern Province)**

Children with DS are always hungry, and they want to eat after every short period. Whenever you have food, you can give them because they are always hungry. **(IDI, Male participant, Kigali City)**

#### 3.2.2 Theme 2: Parental Motivation for Feeding Children With DS

The participants mentioned that despite the challenges of feeding children with DS, various motivating factors encouraged them to ensure their children were well‐fed. The key subthemes that emerged include respect for the child as a creation of God and desire for them to reach their full potential, and witnessing the tangible growth of the child.

##### 3.2.2.1 Respect for the Child as a Creation of God and Desire for Them to Reach Their Full Potential

Participants mentioned that love and respect for a child as a creation of God motivated them to ensure their children were well‐fed. They noted that accepting what God gives them and the love expressed toward their children by different individuals are key motivating factors for feeding their children appropriately. Participants also expressed their belief that their children could reach their full potential if they are well fed.

Something that inspires me to take good care of my child with trisomy 21; first, I have to love him and respect him as a creature of God that he has given me. In our belief, what God gives you, you have to accept and treat him like other children. Also, my child is funny and loving. he is loved by everyone, so it inspires me to care about him. **(FGD, Male participant 1, Kigali City)**

I wanted to see my child grow and I had to try to prevent her from everything that can inhibit her growth. These children require many things, it also requires you to work hard to get what they need, otherwise, you can become a beggar. **(IDI, Male participant, Kigali City)**

##### 3.2.2.2 Witnessing the Tangible Growth of the Child

Some participants mentioned that witnessing their child's growth despite the initial despair was a key motivating factor. They feel amazed to see their children walking or sitting, milestones that were not anticipated in the past.

The reason that motivates me is how the child is now; it now amazes me even though it was difficult then. The child could neither walk nor sit, it was just lying on the bed, but now it gives me the morale to watch the child walk, accompanying his relative to school, and coming from school hungry, it gives me the morale to see what God has done. **(IDI, Female participant, Kigali City)**

#### 3.2.3 Theme 3: Support Systems and Resources for Feeding Children With DS

Different support systems and resources are required for feeding children with DS, given the chronicity and complexity of the condition. Discussions with participants in both in‐depth individual interviews and focus groups revealed two subthemes: “support from the community, government, and healthcare providers” and “family support in caregiving to children with Down syndrome.”

##### 3.2.3.1 Support From the Community, Government, and Healthcare Providers

Most participants in the interviews reported that the primary support they received was from healthcare facilities, which provided essential resources such as food supplements for malnourished children and guidance on caring for children with DS. However, a few participants mentioned that they did not receive any support while dealing with the condition of their child.

The first time I came here at the healthcare facility, they checked the child, and he had about 4 kg. They first gave me porridge flour, and I went home and cooked it for him. When I came back, they found that there was a slight increase in his weight and gave me more supplements for malnourished children. **(FGD, Female participant 7, Eastern Province)**

When healthcare providers started teaching us how to take good care of our children, they told us to be careful and avoid starting by giving them soft foods. I started with plantain and Irish potatoes. **(FGD, Female participant 2, Eastern Province)**

No support received regarding the care of our child with DS. After I got a referral to go to a referral hospital, I tried to go to the district office to see whether I would get some support, but they have not yet answered me. **(IDI, Female participant, Kigali City)**

I did not receive any support that would help me feed my child as required. I give to the child what I can afford. **(FGD, Female participant 5, Eastern Province)**

##### 3.2.3.2 Family Support in Caregiving to Children With Down Syndrome

During the analysis of interviews with various participants, it was found that some family members, particularly husbands, actively supported caregiving for their children, with some even quitting drinking alcoholic beverages after having a child with DS.

The father of this child always supports me for different needs of the family, but I am the one who takes care of her every time. **(IDI, Female participant, Kigali City)**

I was lucky not to face conflicts in the family because of having a child with DS. My husband was a drunker when I gave birth to this child with DS, but he decided to quit alcohol when he saw the situation, and we worked together to raise her. **(IDI, Female participant, Kigali City)**.

#### 3.2.4 Theme 4: Challenges Related to Feeding Children With DS

The analysis of interviews with caregivers revealed that the majority experienced three main subthemes of challenges: (a) financial influences on food choices, (b) anatomical anomalies contribute to feeding difficulties, and (c) lack of support from male partners.

##### 3.2.4.1 Financial Influences on Food Choices

The majority of participants indicated that financial constraints negatively affected how they fed their children with DS, as they could not afford certain nutritious foods commonly available in the market, such as meat, fish, eggs, and fortified cereals. Additionally, caregivers found it challenging to select the right foods that support overall growth and development, particularly for children with DS who may have difficulties or increased nutritional needs. To mitigate these challenges, they opted for more affordable, locally available alternatives with almost similar nutritional value, such as beans instead of meat, small dried fish commonly consumed in Rwanda known as “indagara,” porridge made from sorghum or millet, and sweet potatoes.

The challenge lies in the lack of sufficient financial resources, particularly evident in high market prices, making it difficult to afford items like meat. **(IDI, Male participant, Kigali City)**.

Since sometimes we are required to use expensive things, we try to check among the 3 kinds of foods, namely energy‐giving foods, body‐protective foods, and body‐building foods. Among those 3 kinds of foods, let us say if I do not get meat, I ensure to get cheap small fish and vegetables from our neighbor. Those are the strategies that some of us use, and we see results day and night. **(FGD, Female participant 4, Eastern Province)**

##### 3.2.4.2 Anatomical Anomalies Contribute to Feeding Difficulties

Anatomical defects negatively impact the feeding of children with DS, as they often cannot eat on their own or do so easily, making it difficult for caregivers to ensure they get enough food for proper growth.

For me, the challenge I encountered is that the child cannot take in the food on his own. That's what makes it difficult for me. **(FGD, Female participant 3, Eastern Province)**

There are challenges. First, when I gave him porridge, he couldn't take it by himself. I had to sit and pour it into his mouth and see that he was full or refused and spit it out. **(FGD, Female participant 5, Eastern Province)**

##### 3.2.4.3 Lack of Support From Male Partners

Even if some participants reported receiving support from family members, many pointed out a lack of support from their spouses, particularly male partners. The responsibility of raising a child with DS sometimes triggered conflicts within families. In several cases, husbands either stopped their support, abandoned their families, or harassed their partners. For example, one participant shared how her husband left after the child's diagnosis, accusing her of infidelity and refusing to help with the household and farming. Another participant described how her husband abandoned her just months after childbirth, leaving her to care for the child alone despite ongoing medical needs.

After the confirmation of the problem at a district hospital, my husband stayed at home for few days and then left home. He said that he cannot cultivate alone while his wife sits at home caring for a child with DS. He accused me of bringing a child from another man, and he was asking me to take the child back to that man who is her father. **(IDI, Female participant, Eastern Province)**.

My husband does not support me. He abandoned me. Right after childbirth, now the child is 6 years old, but he left when the child was 3 months old when I had started to go to the referral hospital for care. **(IDI, Female participant, Kigali City)**.

## 4 Discussion

It has been observed that children with DS experience delays in being introduced to complementary feeding. Participants mentioned that anomalies often seen in children with DS may impede their chewing and swallowing abilities, contributing to delays in being ready for feeding. Caregivers highlighted the increased frequency of feeding required for these children compared to those without DS, as a means to address and compensate for these feeding challenges. These findings are supported by another study, which revealed that feeding difficulties are predominant in children with DS and affect all three phases of swallowing, with the greatest difficulties occurring with solids, followed by liquids [13]. Another study has shown that children with DS may eat more slowly than typically developing children due to oral‐motor and chewing difficulties, often requiring more time to complete meals [21]. All these findings may explain the adaptive strategies identified in this study, where caregivers increased feeding frequency to ensure adequate nutritional intake for their children.

A scoping review further highlighted these feeding challenges, showing that caregivers of children with DS face difficulties in introducing complementary feeding, with solid foods often introduced later than World Health Organization (WHO) recommendations [11]. The WHO recommends introducing complementary foods at 6 months of age while continuing breastfeeding. Infants should be fed two to three times a day between 6 and 8 months, increasing to three to four times daily from 9 to 24 months, with one and two nutritious snacks added for children aged 12–24 months. As the child grows, food consistency and variety should gradually increase, starting with pureed or mashed foods and progressing to finger foods by 8 months. By 12 months, most children can eat the same foods as the rest of the family, with an emphasis on nutrient‐dense options, including animal‐sourced foods like meat, fish, eggs, and dairy [22].

Several factors contribute to this delay, including delayed oral motor development and chewing skills, parental concerns about choking, and early‐life surgical or medical interventions, which can disrupt eating development and lead to food aversions [11]. This highlights the need to consider the special needs of these children and to be prepared for potential difficulties or delays in starting complementary feeding. Healthcare providers involved in the care of these children and the development of nutrition policies for this population should consider their unique needs, ensuring they receive food that aligns with their tolerance, growth, and developmental status.

Despite the challenges caregivers face, they expressed that their motivation stemmed from love and respect for their children as creations of God. They believed that if their children were well‐fed, they could reach their full potential, which could only be achieved through proper nutrition. Another motivating factor was the ability to witness tangible growth as their children developed, which made them feel a responsibility to feed them more in order to continue witnessing these changes, even if it differed from their initial hopes. These findings are consistent with a study by Brantley et al., which found that caregivers often experience heightened stress due to concerns about their children's food intake. As a result, they tend to feed their children more frequently to ensure they receive adequate nutrition [14]. This underscores the motivation caregivers feel to meet their children's nutritional needs, even when faced with feeding challenges.

The majority of participants reported receiving tangible support from healthcare facilities in collaboration with government and community entities, whereas only a few mentioned not receiving any support. This finding aligns with another study that showed healthcare professionals are increasingly providing the necessary care to children with DS, reflecting growing societal acceptance of DS [23]. It highlights a progressive understanding of DS within the Rwandan community, especially among healthcare providers, suggesting reduced discrimination in the care of children with special needs and the likelihood of ongoing support.

Finally, this study explored the challenges associated with feeding children with DS. One of the key challenges reported by participants was financial constraints, which hinder caregivers from providing appropriate and diverse foods for their children.

These findings are consistent with previous studies, which have highlighted financial limitations as a common barrier to adequate child nutrition, particularly in low‐income countries such as Rwanda where this study was conducted. Feeding difficulties related to limited financial resources are not exclusive to families of children with DS but are also prevalent among families of children without disabilities [24, 25]. Another challenge reported by participants was the presence of anatomical anomalies in children with DS that interfere with proper feeding. These findings align with those of other studies, which also highlighted challenges related to anatomical defects in children with DS, noting that such defects negatively impact their feeding [7, 11]. This finding highlights the need for comprehensive assessments of children with DS during healthcare visits, as well as the importance of educating caregivers on how to adapt feeding strategies based on each child's anatomical anomalies and feeding abilities.

Participants also reported the lack of adequate support from family members, particularly male partners, as another challenge. Many women reported that their husbands abandoned the household after the birth of a child with DS, which resulted in little to no support for the mothers in raising their children with DS. This challenge contrasts with findings from a study conducted in the United States, which reported significantly lower divorce rates among families of children with DS compared to those with children without disabilities [26]. This discrepancy may be attributed to cultural and social differences between the study settings, where families in developed countries such as the United States may have greater acceptance and support systems for raising a child with a disability like DS compared to those in developing countries like Rwanda. This highlights the need to increase public awareness in Rwanda about DS and to provide counseling and support to family members to help them cope with the situation.

## 5 Strengths and Limitations

The strengths of this study arise from its unique focus, as DS has not been previously explored, particularly in Rwanda. By combining IDIs and FGDs, we gained a comprehensive understanding of the feeding patterns of caregivers for children with DS. However, the findings of this study cannot be generalized due to the limited number of study settings and the inherent nature of qualitative research, which does not aim for generalizability of the study settings [27]. Another limitation of this study is the inability to conduct member checking, a procedure in which researchers return to participants to confirm whether the interpretation of their responses accurately reflects their views. As a result, there is a possibility that some of the participants’ thoughts or experiences may not have been fully or correctly captured. Finally, the ages of the children with DS were not collected. Feeding experiences may vary across developmental stages, and having this information could have enhanced the transferability of the findings. Future studies should address this gap.

## 6 Conclusion

The current study revealed diverse experiences among caregivers, highlighting that children with DS often have multiple health issues and delays that contribute to delayed readiness for complementary feeding. Despite these delays, caregivers expressed motivation to feed their children, viewing it as their responsibility and believing their children can still reach their full potential. Some of the coping strategies reported by participants included making multiple feeding attempts throughout the day and patiently introducing different types of food gradually, based on the child's tolerance. Participants also reported insufficient support from family members, particularly male partners, as well as financial constraints that hindered their ability to provide adequate nutrition. However, a few noted that their partners had even quit drinking alcoholic beverages to support them. These findings highlight the need for further studies to assess family involvement in the care of children with DS, explore coping strategies in the absence of support, and identify possible interventions to enhance family engagement in caregiving.

## Author Contributions

Joselyne Rugema, Eric Matsiko, Donatilla Mukamana, and Leon Mutesa had substantial contributions to the conception of the work and worked on the methodology of the study. Joselyne Rugema and Vedaste Bagweneza collected data. Joselyne Rugema, Eric Matsiko, Vedaste Bagweneza, Donatilla Mukamana, and Leon Mutesa performed analysis. All authors wrote the original draft of the manuscript, participated in final approval of the version to be published, and made a review and editing of the final manuscript. Eric Matsiko, Donatilla Mukamana, and Leon Mutesa did supervision.

## Conflicts of Interest

The author declares no conflicts of interest.

## Funding

This research was funded with support from the ELMA Foundation (Grant No. 347/UR‐CMHS/2024) through the School of Nursing and Midwifery, College of Medicine and Health Sciences, University of Rwanda.

## Data Availability

The data that support the findings of this study are available on request from the corresponding author. The data are not publicly available due to privacy or ethical restrictions.
